# Stable, neuron-specific gene expression in the mouse brain

**DOI:** 10.1186/s13036-023-00400-5

**Published:** 2024-01-16

**Authors:** Osama Ahmed, Kingsley M. Ekumi, Francesco V. Nardi, Gulimiheranmu Maisumu, Khaled Moussawi, Eric D. Lazartigues, Bo Liang, Abraam M. Yakoub

**Affiliations:** 1 Department of Medicine, Harvard Medical School, and Brigham and Women’s Hospital, Boston, MA USA; 2 Biomedical Engineering Program, University of North Dakota, Grand Forks, ND USA; 3 Department of Psychiatry, University of Pittsburgh, Pittsburgh, PA USA; 4 Cardiovascular Center of Excellence, Louisiana State University Health Sciences Center, New Orleans, LA USA; 5 Southeast Louisiana Veterans Healthcare System, New Orleans, LA USA

**Keywords:** Gene expression, Brain, Gene therapy, Brain disease, Genetic engineering, Gene delivery

## Abstract

Gene delivery to, and expression in, the mouse brain is important for understanding gene functions in brain development and disease, or testing gene therapies. Here, we describe an approach to express a transgene in the mouse brain in a cell-type-specific manner. We use stereotaxic injection of a transgene-expressing adeno-associated virus into the mouse brain via the intracerebroventricular route. We demonstrate stable and sustained expression of the transgene in neurons of adult mouse brain, using a reporter gene driven by a neuron-specific promoter. This approach represents a rapid, simple, and cost-effective method for global gene expression in the mouse brain, in a cell-type-specific manner, without major surgical interventions. The described method represents a helpful resource for genetically engineering mice to express a therapeutic gene, for gene therapy studies, or to deliver genetic material for genome editing and developing knockout animal models.

## Introduction

Multiple methods for transgene delivery to the mouse brain have been devised, such as retro-orbital injection through the retina, intravenous (IV) injection, *in-vivo* electroporation, intracerebroventricular (ICV) stereotaxic injection, and other methods (Table 1). Retro-orbital injection leads to gene expression in the brain and the retina [1, 2], but it suffers drawbacks, as the method is very invasive to the pups and may result in eye damage. IV injection of a virus through the tail vein usually requires larger volumes of the virus, and the targeted transgene expression in the brain is very low as the virus diffuses to other tissues before reaching the brain [3]. However, IV injection of adult mice with the adeno-associated virus (AAV)-PHP.eB has shown widespread transgene expression in the brain [4–6]. *In-vivo* electroporation method requires complex optimization. Finding the optimal voltage for widespread delivery in the brain is tedious, and, in some cases, requires germline modifications and complex surgical procedures, especially for *in-utero* electroporation [7–9]. Local stereotaxic injection of virus into the brain is widely used in adult mice [4, 5], but the extent of transgene expression is highly restricted to the injection area [10]. As an alternative, stereotaxic ICV injection provides an approach to deliver virus directly into the brain of mice, including neonatal mice, by taking advantage of the ventricular system anatomy and cerebrospinal fluid (CSF) circulation for widespread gene expression across the mouse brain [4, 5, 11–13]. Other methods for transgene delivery into the mouse brain without the use of stereotaxic instrumentation are the transverse sinus and intravascular injection methods that use less time with minimal equipment, but require specialized handling skill, and often misplacement of the micropipette or needle can lead to damage of the blood–brain barrier [14, 15].

**Table 1:** Comparison of methods used for gene delivery to the mouse brain

Methods for transgene delivery into the mouse brain					
Gene delivery method	**Routes**	**Expression regions**	**Advantages**	**Disadvantages**	**References**
Retro-orbital injection	Retina	Retina and brain	Direct route to the brainbypassing the BBB; efficient and effective for delivering drugs	Disruptive to the pups; potential vision damage; possible anesthesia's systemic effect; special training on anaesthetization	[1, 2]
Intravenous injection	Tail vein	Brain and other tissues	No special equipment needed	Injected substance needs to be able to cross the BBB; larger volumes of virus needs; low transgene expression; diffusion to other tissues; laborious to perform; high failure rate	[3]
In-vivo electroporation	Ventricle/desired brain parenchyma	Whole brain or specific brain region	Direct delivery of DNA; strong expression of transgenes; rapid and efficient	Need optimization; specialized voltage for widespread delivery; high cost; germline modifications; surgical procedures	[7–9]
Intravascular injection	Temporal facial vein	CNS and peripheral organs	Stable and global transgene expression; efficient; minimal stress and quick recovery	Specialized handling skill; possible damage to BBB; injected substance needs to be able to cross the BBB	[15]
Transverse sinus	Transverse sinus	Whole brain	Efficient; fast and simple; less equipment	Specialized handling skill; not enough applications	[14]
Intracerebroventricular injection	Ventricular system	Whole brain	Can be adapted for cell type-specific expression; direct delivery into the mouse brain; no need to cross the BBB; cost-effective; rapid; no surgery needed; no undesirable side effects in neonatal mice; quick recovery in neonatal mice	Non-uniform expression across the brain	[11, 16, 17] Current study

*BBB* Blood–brain barrier, *CNS* Central nervous system

Transgene delivery with stereotaxic ICV AAV injection in mouse pups results in expression patterns that are maintained for up to one year in most brain regions [11, 16, 17]. It provides stable and widespread transgene expression in the mouse brain, if performed on neonatal mice at postnatal day 0 (P0) within 3–6 h of birth, as the brain parenchyma at this age allows the unrestricted dissemination of the virus resulting in global expression in the mouse brain [18]. ICV injection of P1 pups with either AAV-PHP.eB or AAV9 (under the cytomegalovirus (CMV), ubiquitous, promoter) showed transgene expression in multiple brain regions, but with a transduction efficiency of ~ 15–20% of the cells [6]. A major benefit of the ICV route is that the injected virus can enter directly into the central nervous system (CNS) without the need to cross the blood–brain barrier [13]. This procedure is safe, as the mice recover quickly, without undesirable side effects.

**Fig. 1:**
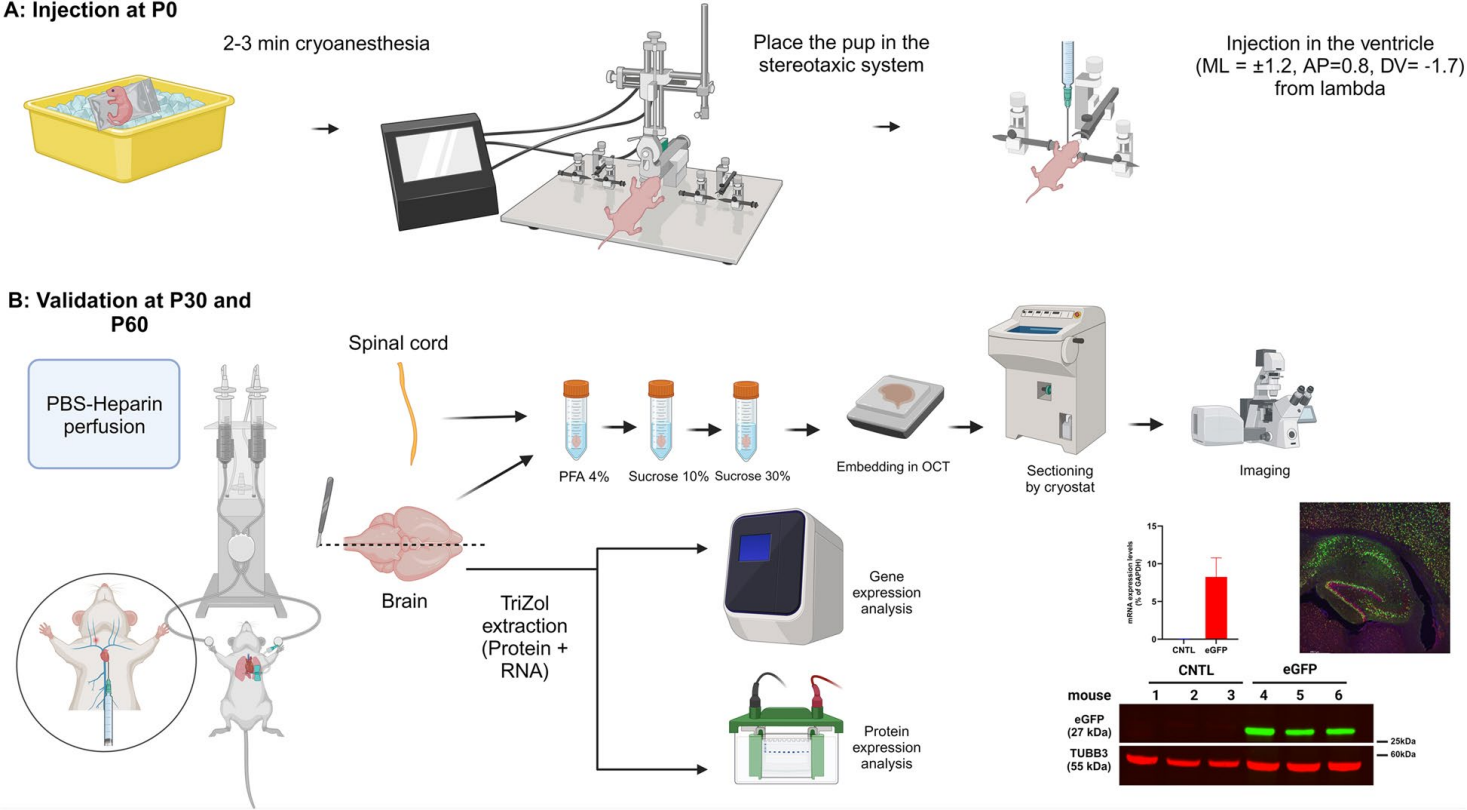
Graphical summary outlining the entire procedure for gene delivery and validating its expression. **A** pup injection with the transgene-expressing AAV9 using stereotaxic ICV coordinates at P0. **B** Molecular and cellular approaches to validate the gene expression in the adult mouse brain and spinal cord. “Created with BioRender.com”

Here, as outlined in the graphical summary (Fig. 1), we describe a rapid, simple, and cost-effective method of gene delivery to the mouse brain using stereotaxic ICV injection for global (brain-wide) transgene delivery in a cell-type-specific manner (using neurons, as an example cell type in this study). We use an AAV9 vector with neuronal tropism [3, 19], under the control of the human synapsin1 (Syn1) gene promoter to ensure the neuron-specific transgene expression. We validate the transgene expression by quantitative real-time reverse-transcription polymerase chain reaction (qPCR), immunoblotting, and immunohistological approaches and provide a detailed explanation of these procedures.

### Experimental design

**Fig. 2:**
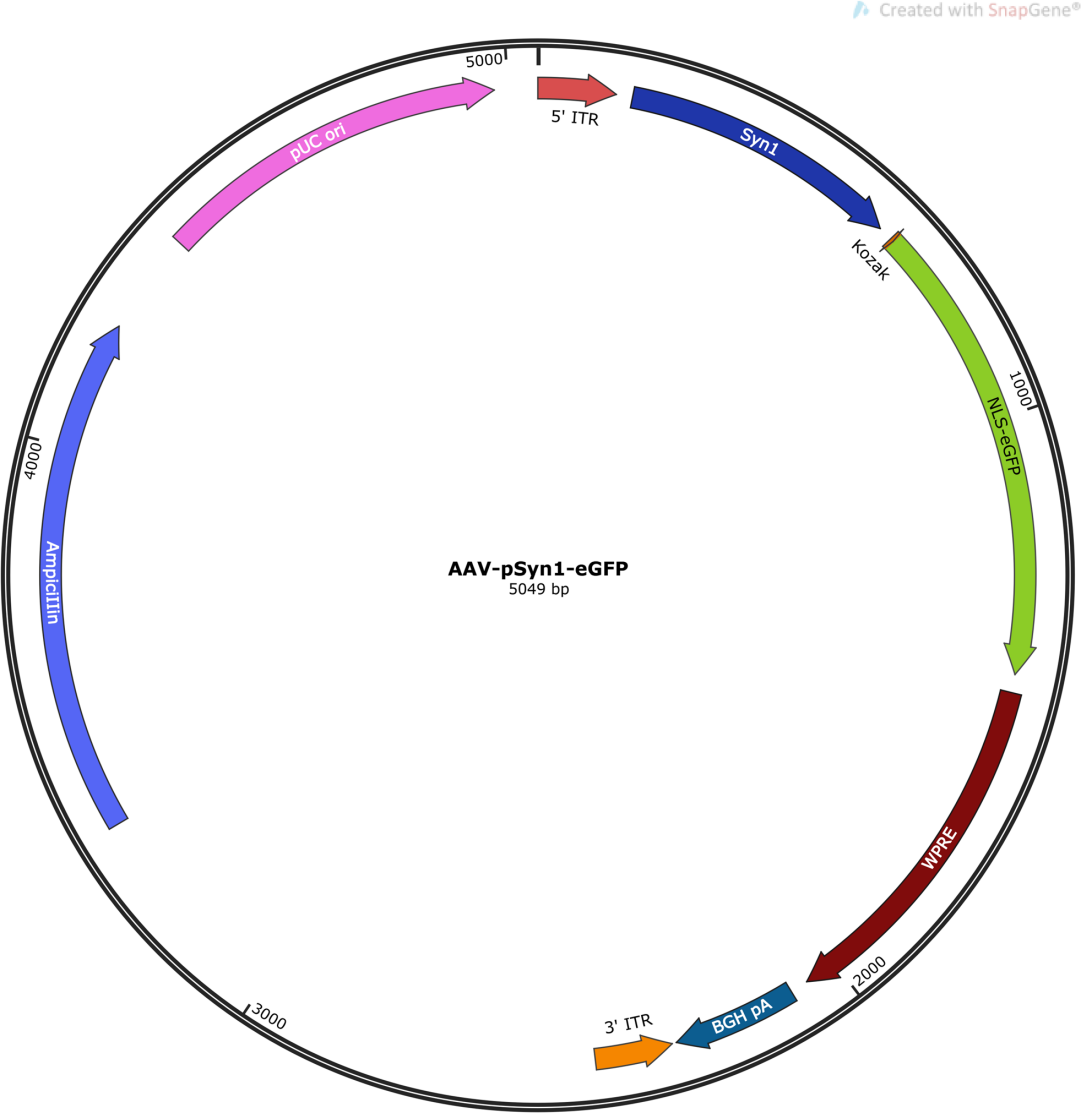
Designed plasmid used to generate the AAV9 for this study. AAV9 expressing the construct encoding NLS-eGFP driven by the human synapsin1 (Syn1) promoter. ITR: inverted terminal repeats; Syn1: Synapsin 1 promoter; Kozak: Kozak sequence; NLS-eGFP: nuclear localization signal-enhanced Green Fluorescent Protein; WPRE: Woodchuck Hepatitis Virus (WHP) Posttranscriptional Regulatory Element; BGH pA: bovine growth hormone polyadenylation signal; Ampicillin: ampicillin resistance gene; pUC ori: pUC origin of replication

The graphical summary (Fig. 1) summarizes the flow of the procedure from the day on which the pups are born to the validation of gene expression in the mouse brain. The plasmid used to generate the AAV9 for this study (Fig. 2) expresses nuclear localization signal (NLS)-eGFP driven by the human Syn1 promoter. The sequence region of the human Syn1 promoter used encompasses the 469 bp upstream of the Syn1 open reading frame, and has shown specificity to target neurons [20].

## Materials and methods

### Animals

Timing is particularly important for this procedure, as ICV injection in neonatal mice brain requires the skull and skin to be thin in order to avoid surgery or drilling through the skull. This injection is best achieved within 3–6 h of birth, as the procedure becomes more difficult and irritating to the mice as they get older. Mouse pups within 3–6 h of birth were used for injections. For this study, wild-type C57BL/6J were obtained from the Jackson Laboratory (Bar Harbor, ME). All procedures were performed in accordance with the respective institutional committees guidelines and approvals. Mice were housed per standard guidelines, with light–dark photoperiod maintained at 12:12 h in a controlled environment temperature of 23 ± 1 ℃ and relative humidity of 55 ± 5% at the animal housing facility.

### Materials

- pSyn1-eGFP-NLS virus (custom-designed and produced; Fig. 2); titer: 1.76 × 10^13^ GC/ml.
- 10X PBS (1610780EDU, BioRad)
- 10X TBS (1706435EDU, BioRad)
- Tween-20 10% Solution (1610781EDU, BioRad)
- Ketamine (VINB-KETO-70121, Covetrus)
- Xylazine (AH0006P, Rampun)
- Heparin (H3393-100KU, Sigma)
- Syringe (701RN, Hamilton)
- Needles (Customized part #7803–07, Hamilton, 30G/15 mm/point 4/20° angle)
- VANNAS spring scissors (S11002-08, RWD)
- Small spatula
- 4% paraformaldehyde (J19943.K2, Life Technology Corporation)
- Trizol reagent (15,596,018, Life Technology Corporation)
- Ethanol 200 Proof (C933N40, Thomas Scientific)
- Isopropanol (BP26181, Thermofisher Scientific)
- First-strand cDNA synthesis master mix 4X (MB6008, ScienCell)
- Primers (IDT, see Table 3)
- Powerup SYBR Green Master Mix (A25778, Applied Biosystems)
- PCR plates reader (Thermofisher Scientific)
- Pipettes and tips
- Guanidine-HCL (G4505, Sigma)
- Urea (U0631, Sigma)
- SDS (151–21-3, Bioworld)
- PBS-T (0.1%Tween-20 in PBS)
- 1% Casein in PBS or 5% non-fat milk in PBS (M-0842, Labscientific)
- O.C.T. compound (4583, Sakura)
- DAPI-prolonged diamond in mounting medium (P36962, Invitrogen)
- 4X Laemmli sample buffer (1,610,747, BioRad)
- Halt protease and phosphatase inhibitor cocktails (PI78446, Thermofisher scientific)
- Primary and secondary antibodies (see Table 2)
- PM2500 protein marker (PM25002112201-1, ExcelBand)
- MES-SDS running buffer (NP0002, Invitrogen)
- HyPure Molecular Biology Grade Water (SH30538.03, VWR)
- Chloroform (LC130402, LabChem)
- MicroAmp Optical adhesive film (4,311,971, Applied Biosystems)
- Superfrost Plus Microscope Slides (22–037-246, Fisherbrand)
- NuPAGE 4–12% Bis–Tris Gel (NP0335B0X, Invitrogen)
- iBlot 2 NC Mini Stacks (IB23002, Invitrogen)
- Triton X100 (97,063–864, VWR)
- 2-Mercaptoethanol (M3148, Sigma)
- 15 ml and 50 ml falcon tubes
- 1.5 ml eppendorf tubes
- Surgical fine forceps (RWD Life science)
- Surgical scissors (RWD Life science)

**Table 2:** Primary and secondary antibodies used in this study

Antibody	Catalog	Application	Dilution
Rabbit eGFP	Novus, NB600-308	Immunoblotting/immunofluorescence	1:1000
Mouse Tuj1	Biolegend, 801,202	Immunoblotting	1:2000
Mouse NeuN	Abcam, ab104224-1	Immunofluorescence	1:1000
IRDye 800 CW	Licor, 926–32,211	Immunoblotting	1:5000
IRDye 680 CW	Licor, 926–68,070	Immunoblotting	1:5000
AlexaFluor 488 goat anti-rabbit	Invitrogen, A11034	Immunofluorescence	1:2000
AlexaFluor 633 goat anti-mouse	Invitrogen, A21052	Immunofluorescence	1:2000

### Antibodies and primers

The primary and secondary antibodies used for immunoblotting or immunohistochemical analyses, and the primer sequences used for the qPCR assays are outlined in Tables 2–3.

**Table 3:** Primers for qPCR used in this study

**Primers**	Sequences (5'—3')
eGFP-F: eGFP-R:	GAG CTG AAG GGC ATC GAC TT CTC GAT GTT GTG GCG GAT CT
GAPDH-F: GAPDH-R:	ACC CAG AAG ACT GTG GAT GG CAC ATT GGG GGT AGG AAC AC

### Equipment

- Biosafety cabinet
- Automatic stereotaxic instrument (71,000-M, RWD Life science)
- Ice maker
- Mouse cages
- Neonatal mouse stage (68,030, RWD Life science)
- T100 Thermal Cycler (1,861,096, BioRad)
- CFX Connect Real-Time PCR cycler (1,855,201, BioRad)
- Tissue homogenizer (15,340,169, FisherBrand)
- Refrigerated microcentrifuge (5430R, Eppendorf)
- Nanodrop machine (13–400-518, Thermofisher Scientific)
- Leica DMi8 Thunder Imager (Leica Microsystems)
- Cryostat (FS800A, RWD Life Science)
- Lab Water Bath 2340 (Thermofisher Scientific)
- Odyssey CLx Imaging System (9140, LICOR)
- Perfusion system (IV 4140, Braintree Scientific)
- Western blot gel tank (A25977, Thermofisher Scientific)
- Multiskan FC microplate photometer (51,119,100, Thermofisher Scientific)
- iBlot 2 Dry Blotting System (IB21001, Thermofisher Scientific)
- MyVOLT Touch (E2301, Accuris)
- MiniPlate spinner (C1000, Labnet)
- Standard analog Shaker (89,032–092, VWR)
- Legato 130 Single Syringe I/W Nanoliter (LEGATO 130, RWD Life science)

### Software

- GraphPad prism (V9.4.1)
- Excel (365)
- NASX navigator
- ImageJ
- Adobe illustrator
- BioRender
- LAS X Thunder
- Image Studio

### Recipes

1. Preparation of Ketamine/Xylazine working stock

To prepare 1 ml: 175 µl ketamine (100 mg/ml) + 25 µl xylazine (100 mg/ml) + 800 µl sterile dist. water.

2. Preparation of blocking buffer for IHC blocking

To prepare 220 ml: 4 ml of 100% animal goat serum + 880 µl Triton X100 + 22 ml 10X PBS (continue with dist. water to 220 ml).

3. Preparation of PBS/Heparin

To Prepare 1000 ml: Add 1 ml of 10,000 Unit Heparin to 1000 ml of 1X PBS.

4. Preparation of permeabilization buffer for IHC

To prepare 500 ml: 5 ml of 100% animal serum + 2 ml Triton X100 + 50 ml 10X PBS (continue with dist. water to 500 ml).

### Procedures and results

#### Anesthesia and stereotaxic injection of pups

Before starting the surgical procedure, thoroughly clean the surgical table and any equipment that will be used. 70% ethanol suffices for surfaces, while tools are sterilized by autoclaving.

**Fig. 3:**
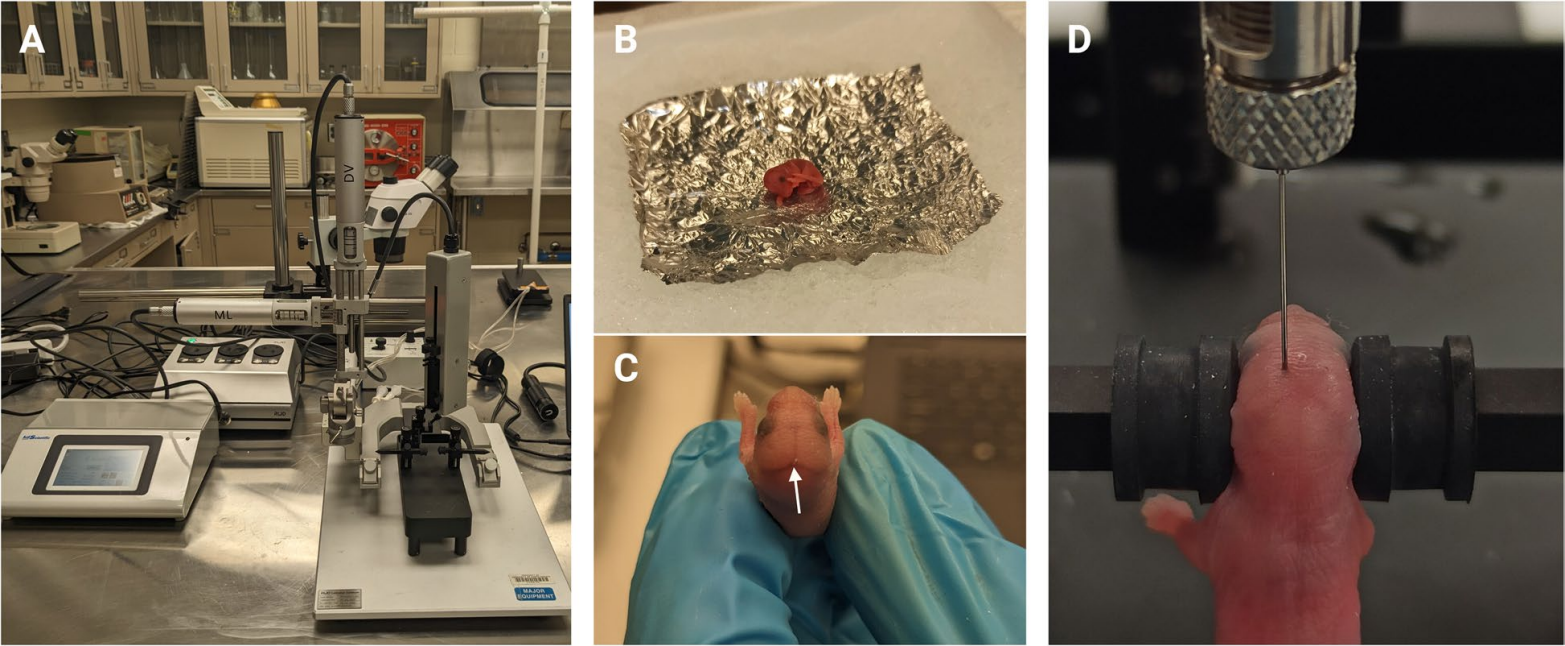
Illustration of the steps involving cryoanesthesia and brain injection. **A** Stereotaxic instrument used for viral injection. **B** For cryoanesthesia, the pup is kept on aluminum foil, and not directly in contact with the ice to avoid skin damage. **C** At P0, lambda (white arrow) and bregma (opposite site) are clearly visible on the surface of the skull. **D** The ICV injection is performed using the following coordinates ML =  ± 1.2, AP =  + 0.8, DV = -1.7 mm from the lambda

- Assemble the stereotaxic instrument by placing the neonatal mouse adaptor on the stereotaxic base.
- Reverse the Jaw holder cuff mouse ear bars.
- Clamp the Legato 130 Infusion/Withdraw Nanoliter Pump syringe holder to the stereotaxic instrument arm (Fig. 3A).
- Place crushed ice in a small bucket and cover the top of the wet ice with cold aluminum foils to protect the pup skin from direct contact with ice.
- Place the pup on top of the aluminum foil for 2–3 minto anesthetize the pup by hypothermia (Fig. 3B). The pup will be fully anesthetized for around 15 min as visualized by slowness in movement.
- Once the pup is fully anesthetized, align the pup on the stereotaxic instrument using the non-rupture rubber ear bars and ensuring lambda is visible and the line between lambda and bregma (see Fig. 3C-D as a reference) is parallel to the Anterior–posterior axis (AP). Note: Avoid squeezing the pup's brain too tight as this will result in pressure back flow and hence variability in the injection steps.
- Place the required volume of the virus/vehicle to be injected in a syringe (Hamilton, 30G) and clamp the syringe on the Legato 130 Infusion/Withdraw Nanoliter Pump syringe holder. Note: 2 µl/hemisphere were used for this study (3.52 × 10^10^ viral particles/hemisphere). The negative control will be injected with the same volume of sterile 1XPBS.
- Set the infusion rate of the Legato 130 Nanoliter pump to 1 µl/minute.
- Gently wipe the anesthetized pup head with a dry cotton bud ensuring lambda is visible.
- Set the syringe needle head to lambda and set lambda as the reference point (Zero).
- Set the stereotaxic coordinate for ICV injection of virus into each hemisphere at coordinates ML (mediolateral) =  ± 1.2 mm, AP (anteroposterior) = +0.8 mm, DV (dorsoventral) = -1.7 mm from lambda.
- Start the Legato 130 Infusion/Withdraw Nanoliter Pump and let the infusion of the virus continue until completed. Once the virus infusion is complete, wait for 5 min to allow complete diffusion of the virus into the mouse brain. After 5 min, withdraw the syringe needle and proceed to the next hemisphere of the brain as from step k.
- Once injection of the virus into both hemispheres is completed, mark the pup with a permanent marker and place the pup back with the biological mother in the home cage and return to the mouse room.
- Once all pups are injected, they are returned to the cage and kept with their mother until weaning day (P21). At P30 and P60 the mice are euthanized, and the brains collected to validate gene expression.

### Monitoring and labeling of the mice

- Ensure that the mother is nursing the pups and observe that the pup is recovering from the hypothermic anesthesia.
- Monitor the injected pup for one hour after the injection and every day for 3 days. Observe for mobility and any slowness in activity.
- The injected mice can be marked by toe numbering (tattooing) after 8–10 days since the permanent marker that was used to label the pups at the time of injection will no longer be visible due to the fur.

### Perfusion and brain extraction

Note: For this study, the mice were euthanized four/eight weeks after the injection.

**Fig. 4:**
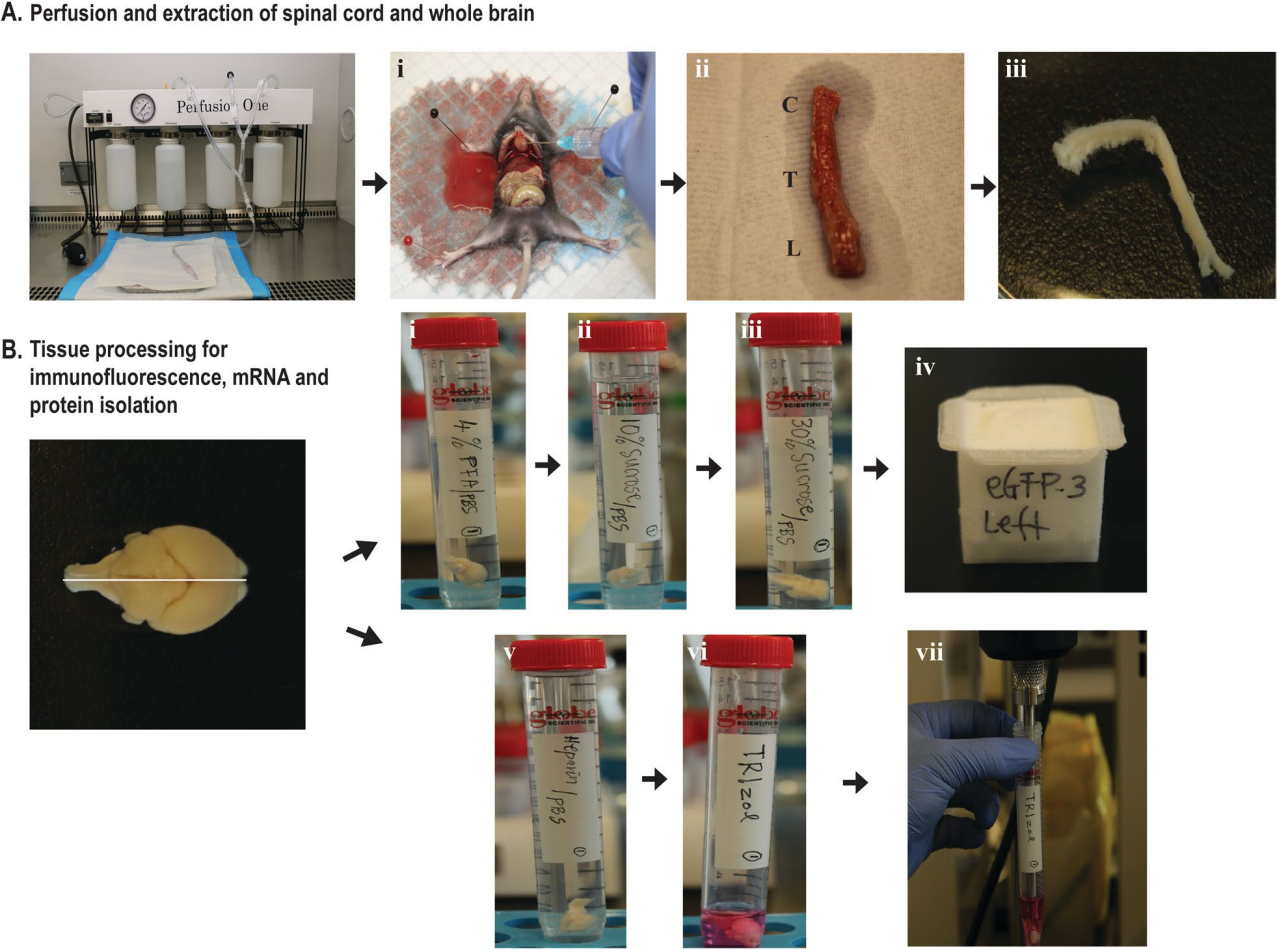
Step-by-step depiction of mouse perfusion, brain and spinal cord extraction and tissue processing. **A** The perfusion apparatus used to perfuse the mouse. (A-i) The perfusion is performed intracardially with ice-cold PBS-Heparin at a constant speed of ~ 200 µl/second. (A-ii and iii) Spinal cord extraction. **B** The brain is cut into two hemispheres. Each hemisphere follows different processing according to the analysis to be performed: one hemisphere is transferred from PFA/PBS 4% to sucrose 10% and 30% (B-i to iii), with every step taking 24 h. The tissue is then embedded in optimal cutting temperature compound (OCT compound) and stored at –80 °C (B-iv); the other hemisphere is transferred to TRIzol and homogenized for mRNA and protein isolation (B-v to vii)

Before starting the surgical procedure, thoroughly clean the surgical table and any equipment that will be used with 70% ethanol. Figure 4 summarizes the following steps for perfusion, extraction, and the subsequent preparation of brain hemispheres for imaging, western blotting, and real time PCR (Fig. 4B-i to vii).

- Assemble the perfusion instrument and prepare the operating table (Fig. 4A).
- Measure the body weight to determine the dose of xylazine/ketamine needed.
- Inject freshly prepared xylazine/ketamine mixture (175 µl ketamine (100 mg/ml) + 25 µl xylazine (100 mg/ml) + 800 µl sterile dist. water) at a dose of 100 µl/20 g body weight through intraperitoneal injection at the lower left or right quadra of the abdomen.
- Place the mouse back in its cage. Wait for 10 min. Assess if the mouse has reached a surgical plane of anesthesia by losing response to toe pinches.
- Place the mouse on its back on the dissection plate. Note: Operate inside a fume hood.
- Grip the skin on the chest with forceps and make an incision using tissue scissors to expose the xiphoid (a piece of arrowhead-shaped white bone).
- Grip the xiphoid with ophthalmic forceps and make lateral incisions beneath the ribcage using tissue scissors to expose the diaphragm and liver.
- Carefully make incisions in the diaphragm along the entire length of the rib cage using fine scissors.
- Carefully tear off the pericardial sac and any other tissues covering the heart using dissecting forceps to provide a clear view of the heart and vessels.
- Secure the heart with dissecting forceps at a steady position and make a snip in the right atrium to let the blood exit.
- Perfuse with ice-cold PBS-Heparin (10 units of Heparin in PBS). Ensure no bubble enters the perfusion system. Note: The perfusion rate is critical in brain dissection and essential to be slow, as the brain tissue can be damaged when using a fast rate and have holes when it is used for imaging.
- Steadily perfuse ice-cold PBS-Heparin at a constant speed of ~ 200 µl/second.
- Perfuse ~ 20–30 ml ice-cold PBS-Heparin for the P30 mouse (Fig. 4A-i). Note: Check whether the tail of the mouse turns white, since that is an indicator of a correct perfusion.
- Remove the needle and unpin the mouse from the surgical station. And then, decapitate the mouse, and remove the skin from the skull, dissect the brain out. Note: Be extremely careful while extracting the brain from the skull, as the brain is very soft and can be easily damaged.
- Wash the brain by adding it into a 15-ml falcon tube containing 10-ml ice-cold PBS and then decant away the PBS.
- Separate the brain into two hemispheres. Transfer one into 4% PFA/PBS to be used for immunostaining and place the other in ice cold heparin-PBS for RNA and protein isolation.

Stopping point: The hemisphere in heparin-PBS can be removed from the solution and stored at – 80 °C to prevent protein and RNA degradation.

### Spinal cord extraction

Note: Before starting the isolation, thoroughly clean the surgical table and any equipment that will be used with 70% ethanol. The tissue processing and immunohistochemistry steps are the same as for the brain hemisphere (see relevant paragraph).

Following brain extraction, the spinal cord was extracted as previously shown [21] with minor modifications. Briefly:

- After brain extraction, the head is removed by cutting at the base of the skull.
- Remove the viscera and make an incision through the abdominal wall muscles and continue laterally to the vertebral column in both directions.
- The ribs are then cut parallel with and close to the vertebral column on both sides, which is finally removed by making a transverse cut at the level of the femurs (Fig. 4A-ii).
- The vertebral column is then transferred into a 10 mm Petri dish filled with 1 × PBS to facilitate the extraction of the spinal cord. The column is carefully cut at the extremities to expose the spinal cord, and using VANNAS spring scissors, an incision is made along the column to expose the spinal cord.
- Finally, the spinal cord is gently detached using a small spatula from the column and transferred into 4% PFA/PBS for immunostaining (Fig. 4A-iii).

### RNA Isolation

Note: Perform all steps at room temperature (20—25℃) unless otherwise stated. The protocol is performed following the manufacturer's instructions for Total RNA isolation using TRIzol reagent with minor changes.

- Add 2 ml of TRIzol reagent per hemisphere in a 15 ml tube.
- Homogenize the samples using a tissue homogenizer at 15,000 rpm for 40 s.

Stopping point: Samples can be stored at 4℃ overnight or at -20℃ for up to a year.

c. Transfer 1 ml of the homogenate into a 1.5 ml Eppendorf tube and store the rest at -20℃.
d. Incubate the homogenate for 5 min to allow complete dissociation of nucleoproteins complex.
e. Add 0.4 ml of chloroform per 1 ml of the homogenate. Securely cap the tube and vortex briefly for 5 s to thoroughly mix the sample.
f. Incubate the sample for 3 min.
g. Centrifuge the samples for 15 min at 12,000 × g at 4℃ to separate the phases of the mixture. Note: The mixture will separate in an aqueous upper phase, a white precipitate interphase and a lower red phenol–chloroform phase.
h. Transfer the aqueous phase solution into a new 1.5 ml Eppendorf tube without touching the white precipitate interphase. This can be done by angling the Eppendorf tube at 45° and pipetting the aqueous phase solution out.

Note: The white-precipitate interphase and the lower red phenol-chloroform phase solution can be stored at -20℃ for protein isolation.

i. Add 0.5 ml of isopropanol per 1 ml of TRIzol used to the collected aqueous phase. Mix briefly by shaking.
j. Incubate the mixture for 10 min at 4℃.
k. Centrifuge the mixture for 10 min at 12,000 × g at 4℃.

Note: Total RNA will form a visible white pellet at the bottom of the Eppendorf tube

l. Discard the supernatant using 1 ml pipette tips being careful not to dislodge the visible white pellet. Keep the pellet.
m. Resuspend the pellet in 1 ml of 75% ethanol per 1 ml of TRIzol used.

Stopping point: The RNA can be stored at the point for one year at -20℃ or for one week at 4℃.

n. Vortex briefly for 5 s, and then centrifuge for 5 min at 7,500 × g at 4℃.
o. Discard the supernatant using a 1 ml pipette tip trying not to take the precipitated RNA.
p. Air dry the sample for 5–10 min at room temperature.
q. Resuspend the RNA pellet using 50–100 µl of ice-cold nuclease-free water, store the RNA sample at -80 ℃.
r. Measure the RNA concentration using a nanodrop instrument.

### Protein isolation

Note: Continue from step-h of the RNA isolation procedure on the same sample.

- Remove any remaining aqueous phase overlaying the white-precipitate interphase and the lower red phenol–chloroform phase solution using a 200 µl pipette tips. Keep the white-precipitate interphase and the lower red phenol–chloroform phase solution.
- Add 0.3 ml of 100% ethanol per 1 ml of the TRIzol homogenate. Cap the tube and mix thoroughly by inverting the tube several times.
- Incubate the sample for 3 min.
- Centrifuge the sample for 5 min at 2,000 × g at 4 ℃.
- Transfer 0.5 ml of the phenol-ethanol supernatant to a new 1.5 Eppendorf tube. Keep the remaining phenol-ethanol supernatant at 4℃.
- Add 0.75 ml of isopropanol to the phenol-ethanol supernatant and mix briefly by inverting the tube several times.
- Incubate for 10 min.
- Centrifuge the mixture for 10 min at 12,000 × g at 4 ℃ to pellet the proteins.
- Discard the supernatant by using 1 ml pipette tips.
- Transfer the remaining phenol-ethanol supernatant from step (e) to the protein pellet and repeat step f-i.
- Wash the protein pellet with 1 ml of wash solution (0.3 M Guanidine hydrochloride in 95% ethanol) by incubating in the wash solution for 20 min.
- Centrifuge the tubes for 5 min at 7,500 × g at 4℃. Discard the supernatant and keep the protein pellet to air dry for 5–10 min.
- Solubilize the protein pellet by adding 300 µl of solubilization solution (1:1 ratio of 1% SDS/TBS and 8 M Urea) and incubate in a water bath at 50℃ overnight.
- Centrifuge the solubilized protein sample for 10 min at 10,000 × g at 4 ℃. Transfer the solubilized protein in a new tube ensuring it is not taken out the insoluble pellets.
- Measure protein concentration using nanodrop instrument or alternatively performing bicinchoninic acid assay (BCA) using an appropriate dilution.
- Store the solubilized protein at -20 ℃ for short term, or at -80 ℃ for long term.

### cDNA synthesis and qPCR analysis

- Using the total RNA isolated from mouse brain, pipette out 1–2 µg of the isolated RNA from each brain and prepare the first strand cDNA synthesis reaction in a total volume of 20 µl in a PCR tube as following, total RNA 1–2 µg, 4 × First strand cDNA synthesis master mix 5 µl, and complete the volume to 20 µl with Nuclease free water.
- Cap the tube and mix by spinning for 20 s in a microcentrifuge.
- Run the reaction in a PCR machine using the following setting: 25 ℃ for 10 min, 45 ℃ for 1 h, 85 ℃ for 5 min and infinite hold at 4 ℃. Note: The synthesized cDNA can be diluted by adding 80 µl nuclease free water to make the cDNA concentration to 20 ng/µl.

### Stopping point: The synthesized cDNA can now be stored at -20 ℃

d. Using the newly synthesized cDNA, prepare the qPCR reaction for a single 20 µl total volume reaction, cDNA 1–2 µl (20–40 ng), 2 × PowerUp qPCR Master mix 10 µl, forward and reverese primer mix (10 µM) 2 µl, and continue the volume to 20 µl with nuclease free water. Ensure that samples are always kept on ice. Prepare a master mix sample for the total number of reactions.
e. Seal the plate using an adhesive film and ensure the plate is well sealed around the edges. Spin the sample for 20 s using a microcentrifuge.
f. Run the reaction in a qPCR machine using the following setting: Activation at 95 ℃ for 10 min, Denaturation at 95 ℃ for 15 s, Annealing/Extension at 60 ℃ for 1 min, repeat the Denaturation and Annealing/Extension for 40 cycles.
g. Analyze the results of mRNA of the gene of interest (A) as percentage of the housekeeping gene (B) using the formula: $\%\boldsymbol{B}= \mathbf{100}\times{2^{\wedge}}^{\left[-\left(\boldsymbol{average}(\boldsymbol{Ct}\;\boldsymbol{of}\;\boldsymbol{A})\;-\;\boldsymbol{average}(\boldsymbol{Ct}\;\boldsymbol{of}\;\boldsymbol{B})\right)\right]}$
h. Represent the results using GraphPad prism or other software by plotting a bar graph of the % of housekeeping gene on the *y-axis* and each brain sample on the x-axis.
i. Use an appropriate statistical test to analyze the significant differences between the control and the gene of interest and set *p-value* < 0.05 to be statistically significant.

### Western blot analysis

- Pipette out 50 µg/lane of protein sample and mix it in a 3:1 ratio with the sample buffer. Note: To prepare the sample buffer, mix 100 µl 2-mercaptoethanol to 900 µl of 4X sample buffer.
- Mix the samples by spinning briefly.
- Heat the sample at 98 ℃ for 5–8 min. Note: Use safe-lock Eppendorf tubes to prevent openings and consequent volume loss.
- While heating the samples, prepare 1X MES-SDS Running buffer and fill the western tank. Cast a 4–12% MES SDS-PAGE gel and rinse each lane using a 200 µl pipette tips by slowly pipetting the running buffer inside each well.
- After heating, keep the samples at room temperature for 2–5 min. Then briefly spin down the samples and load the total volume in the 4–12% MES SDS-PAGE gel. Note: A protein marker is essential to determine the approximate molecular weight of the proteins of interest.
- Close the cassette and run the gel at 150 V for 45 min at room temperature. Run at constant voltage to prevent drastic changes in temperature.
- Transfer the gel to a tray  containing distilled water. Remove the gel from the cassette using the appropriate tools.
- Assemble the iBlot stacks and place the gel for the dry transfer. Note: For this study, a nitrocellulose membrane was used.
- Transfer the gel using the following sequential parameters: 20 V 1 min, 23 V 4 min, and 25 V 2 min.
- Block the membrane using 5% non-fat milk in TBS-T for 1 h at room temperature. Note: To prepare TBS-T, add 250 µl of 10% Tween-20 to 500 ml of 1X TBS. Since Tween-20 is viscous, we suggest cutting a 1000 µl pipette tip using scissors.
- Wash the membrane three times with TBS-T for 5 min.
- Add the primary antibodies in 5% non-fat milk in TBS-T and incubate overnight at 4 ℃ while shaking.
- Wash the membrane three times for 5 min using TBS-T.
- Add the secondary antibodies in 5% non-fat milk in TBS-T and incubate for 1 h at room temperature.
- Wash the membrane extensively three times 10 min each wash. Then wash briefly with only TBS.
- Visualize the proteins of interest on the blot using an imager.

## Tissue processing and immunohistochemistry

### Continue from the section "Perfusion and Dissection" step p

- Keep the collected hemisphere, or spinal cord, in 4% PFA/PBS at 4 °C overnight.
- Wash the hemisphere, or spinal cord, with ice-cold PBS, and then transfer it into 15 ml falcon tube containing 10% sucrose prepared in 1X PBS. Place the tube at 4 °C overnight and wait until the tissue sinks.
- Transfer the hemisphere into 30% sucrose and place it at 4 °C overnight until the tissue sinks.
- Embed the tissue in OCT compound: place the hemisphere, or spinal cord, onto a pre-labeled tissue base mold. Cover the block with OCT slowly and avoid air-bubbles formation, slowly place the base mold containing the block into liquid nitrogen or dry ice till the entire block is frozen completely.
- Store the frozen tissue block at -80 °C overnight before sectioning.
- Transfer the frozen block to a cryostat (make sure the temperature is -20 °C prior to sectioning) and allow the frozen block to reach the cryostat temperature after placing it on the cutting chuck.
- Section the frozen tissue block into the desired thickness (for brain tissue, usually 15–40 µm) and place the sections on double positive slides. Note: It is recommended to keep the slides inside the cryostat (-20 °C) after placing the tissue section on them to avoid tissue damage.

### Stopping point: Slides can then be stored at -80 °C

h. After warming the slides at room temperature, wash them twice with freshly prepared 1X PBS.
i. Draw a circle around the tissue with a hydrophobic barrier pen and let it dry for 2–3 min.
j. Permeabilize the tissue by washing the sections twice, 15 min each, with permeabilization buffer (1% animal serum, 0.4% Triton X-100 in PBS).
k. Block non-specific bindings by incubating the tissue sections with 5% animal serum in PBS-T (0.4% Triton X-100 in PBS) for 1 h at room temperature in a humidified chamber. Note: permeabilization and blocking buffers should be stored at 4 °C, and the animal serum use depends on the species of the secondary antibody.
l. Remove the Blocking buffer by rinsing the slides in 1X PBS twice for 5 min each.
m. Add the primary antibody diluted in 1% animal serum in PBS 0.1% Triton X 100, to the sections and incubate in a humidified chamber at 4 °C overnight.
n. Remove the primary antibody by rinsing the slides in PBS-T for three times, 10 min each.
o. Add fluorescent-conjugated secondary antibody diluted in 1% animal serum in PBS 0.1% Triton X 100 to the slides and incubate at room temperature for 1 h.
p. Remove the secondary antibody by rinsing the slides in PBS-T three times, 10 min each.
q. Tap off excess wash buffer and apply a drop of antifade mounting medium with or without DAPI to the slide. Note: DAPI-prolonged with diamond antifade medium is recommended.
r. Place a coverslip on the tissue section, allow the slides to dry by keeping them in a box at room temperature overnight. The next day store the slides at 4 °C in a box to prevent photobleaching.
s. Visualize using a fluorescence microscope 20X (HC PL FLUOTAR L, PH1), 63X (HC PL APO), FITC (eGFP) Ex: 460–500, DAPI, Ex: 325–375, Far Red (NeuN), Ex: 622–654.

### Validation of gene expression in the mouse brain and CNS

**Fig. 5:**
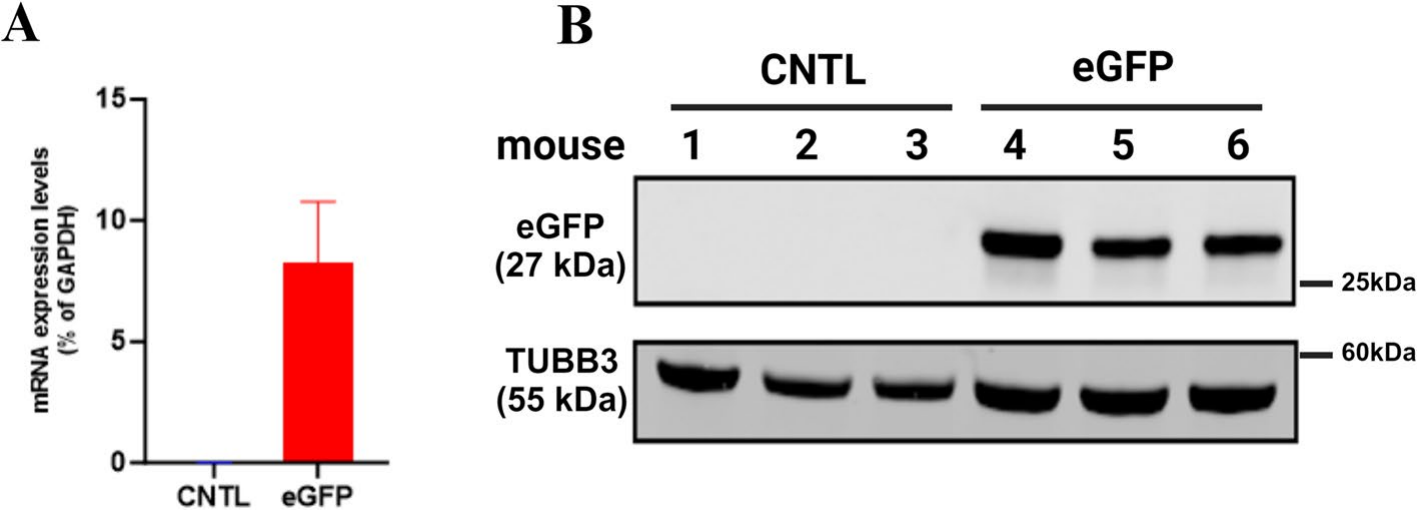
eGFP mRNA and protein are highly expressed in the mouse brain. **A** eGFP mRNA expression levels were determined by qPCR and expressed as % of GAPDH levels; bars represent mean ± SEM, *n* = 3 mice. **B** eGFP protein expression was confirmed using immunoblotting on protein samples from mouse brain lysates. Immunoblotting was performed using anti-eGFP (dilution 1:1000) as the primary antibody, and IRDye-800 CW (dilution 1:2000) as the secondary antibody, with TUBB3 (beta 3 tubulin) employed as the internal loading control. CNTL: Control

**Fig. 6:**
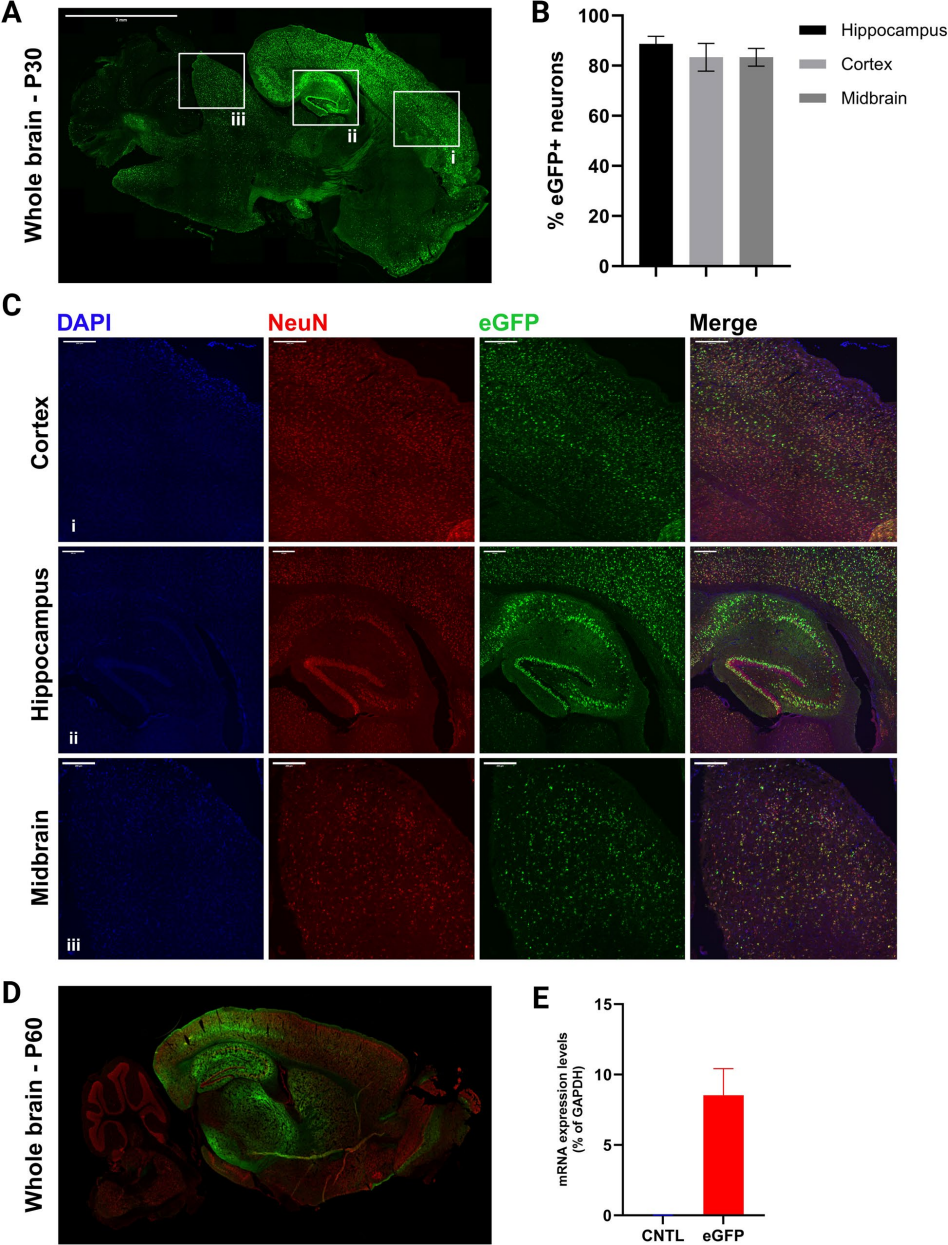
Robust and stable eGFP expression throughout the mouse brain. **A** Immunohistochemistry of P30 mouse brain showing the expression of eGFP in the periventricular regions of the CNS, such as cortex (i) and hippocampus (ii), as well as distant subcortical areas (iii). **B** Quantification of GFP positive neurons in different brain regions (*n* = 3 mice), values are represented as mean ± SEM (**C**) Higher magnification (20x) images of the indicated regions i, ii, and iii in panel (B). **D** Fluorescence microscopical image of a P60 mouse brain. **E** qPCR analysis on a P60 mouse brain mRNA; bars represent mean ± SEM, *n* = 3 mice. CNTL: Control, P30: Postnatal day 30, P60: Postnatal day 60

**Fig. 7:**
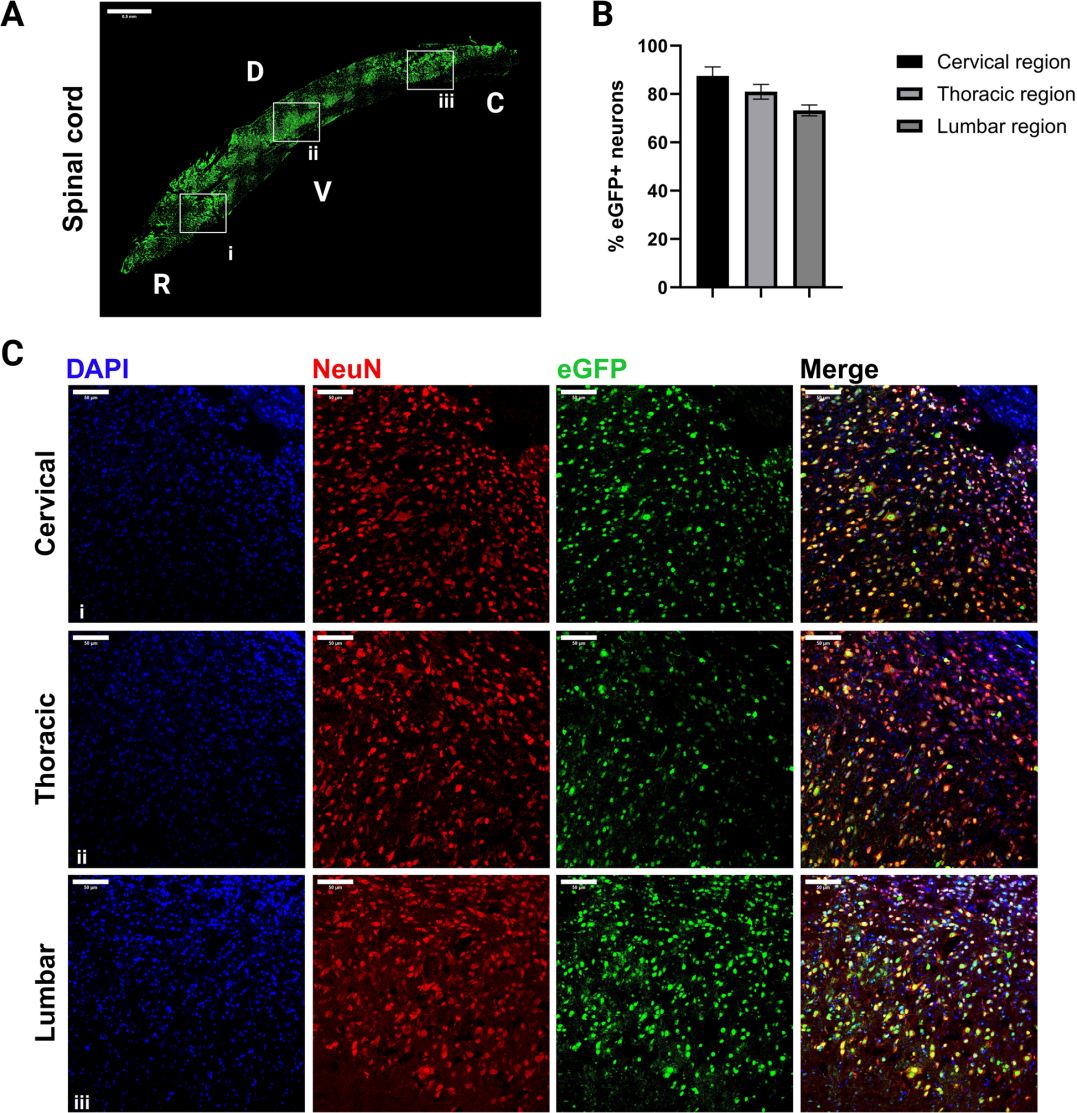
eGFP expression in the spinal cord. **A** Fluorescence microscopical image of a P30 mouse spinal cord; three areas (i, ii, and iii) were arbitrarily chosen for higher magnification images in panel (C). D: Dorsal, V: Ventral, R: Rostral, and C: Caudal. **B** Quantification of eGFP positive neurons in cervical, thoracic, and lumbar spinal cord regions (*n* = 3 mice). Values are represented as mean ± SEM. **C** Higher-magnification (20 x) fluorescence microscopical images of the indicated areas in panel (A). CNTL: Control, P30: Postnatal day 30, P60: Postnatal day 60

Our first goal was to confirm the transgene (eGFP) expression by qPCR and immunoblotting in the injected mice. We found high eGFP expression using qPCR, and then confirmed protein expression using immunoblotting, on samples from P30 mice (Fig. 5). Next, to validate the robust and stable eGFP expression, using immunofluorescence, we found that eGFP expression is widely distributed throughout the mouse brain, with areas close to the ventricle displaying higher eGFP expression. The eGFP gene expression was confirmed in multiple brain regions, including the cortex, the hippocampus, and the midbrain (Fig. 6). For each of these regions, stereological quantification of small regions of interest was manually performed by two independent investigators in a blinded manner using the plug-in “Cell Counter”. (Fig. 6A-C). To further validate the stability of eGFP expression over extended periods, we analyzed brains of P60 mice by immunofluorescence and qPCR and validated sustained expression for at least two months after injection (Fig. 6D-E). Stable gene expression, via AAV injection, over longer periods of time, sometimes up to 10 months, has been reported [16]. Finally, to investigate the extent of eGFP distribution in the CNS, beyond the brain, sagittal sections of the spinal cord of P30 mice were analyzed in the same way. We found that areas close to the brain display higher expression levels of eGFP (cervical region), which tends to slightly decrease in more distant area (toward the lumbar region) (Fig. 7A-C).

### Potential problems and troubleshooting

#### Injection

- If the pup starts moving after the injection in the first hemisphere, the investigator can put it back again on ice for 3–4 min. Usually, the injection should not exceed 5–10 min. It is recommended to avoid keeping the pup in direct contact with the ice to prevent skin damage.
- Avoid placing more pups on ice. Start the anesthesia of one pup after completing the injection of the other.
- Avoid squeezing the pup's brain too tight as this will result in pressure back flow and hence variability in the injection step.
- The injection site may start bleeding after the removal of the needle, if the investigator has not waited for about 5 min to allow complete diffusion of the virus into the mouse brain. Another important reason can be due to injecting with high infusion rate, for this kind of injections it is recommended to restrict to 1 µl/minute.
- Do not return the pup to the mother's cage while some blood drops are on its head after injection, as the mother can act aggressively. If that is the case, gently wipe the blood off the pup head with a cotton dry bud before returning it back to the cage.

### Perfusion and brain dissection

- The perfusion rate is critical, as the brain tissue can be damaged when using a fast rate and present holes when used for imaging.
- Do not stop perfusion until the mouse limbs and tail turn white. Starting the dissection without a complete perfusion can affect the quality of fixation.
- During dissection, make sure that the brain is completely free from all sides before taking it out of the skull. Handle it carefully to avoid tissue disruption.

### Processing of brain tissue—sectioning and staining

- Transfer the brain from one sucrose solution to the other only after it sinks.
- During the tissue embedding, make sure to avoid air-bubbles formation as this can affect the integrity of the brain selection during sectioning.
- It is recommended to place the slide rack directly inside the cryostat after collecting the tissue sections on it, so to prevent tissue damage.
- Permeabilization and blocking buffers should be stored at 4 °C and the animal serum used depends on the species of the secondary antibody.

## Conclusion

We describe how to achieve stable, cell-type (neuron)-specific gene expression in the mouse brain through ICV injection in pups. Using the reporter gene eGFP driven by human Syn1 promoter, we specifically targeted neurons and showed a robust expression in thebrain, for weeks to months after the injection. The gene expression was confirmed through mRNA and protein determination and by immunohistology and immunofluorescence microscopy. Neurons positive for eGFP have been widely detected in several brain regions, including subcortical regions, and in the spinal cord. This protocol represents a simple, fast, and highly adaptable procedure that can be used to deliver genes to the mouse brain.

## Data Availability

The datasets used and/or analyzed during the current study are available from the corresponding author on reasonable request.

## Abbreviations

ICV: Intracerebroventricle
IHC: Immunohistochemistry
CSF: Cerebrospinal fluid
CNS: Central nervous system
SC: Spinal cord
P0: Postnatal day 0
AAV9: Adeno-associated virus 9
qPCR: Quantitative Polymerase chain reaction
Syn1: Synapsin 1
NLS: Nuclear localization signal
PBS: Phosphate-buffered saline
TBS: Tris-buffered saline
SDS: Sodium Dodecyl Sulfate
OCT: Optimal cutting temperature compound
NeuN: Neuronal nuclear protein
